# Conventional Laparoscopy versus Robotic-Assisted Aortic Lymph-Nodal Staging for Locally Advanced Cervical Cancer: A Systematic Review and Meta-Analysis

**DOI:** 10.3390/jcm11123332

**Published:** 2022-06-10

**Authors:** Mariano Catello Di Donna, Vincenzo Giallombardo, Giuseppina Lo Balbo, Giuseppe Cucinella, Giulio Sozzi, Vito Andrea Capozzi, Antonino Abbate, Antonio Simone Laganà, Simone Garzon, Vito Chiantera

**Affiliations:** 1 Unit of Gynecologic Oncology, ARNAS “Civico—Di Cristina—Benfratelli”, 90127 Palermo, Italy; marianocatellodidonna@gmail.com (M.C.D.D.); vigiallo@gmail.com (V.G.); giusilobalbo@gmail.com (G.L.B.); giuseppecucinella@outlook.com (G.C.); giuliosozzi@hotmail.it (G.S.); abbatenino.gyn@gmail.com (A.A.); vito.chiantera@gmail.com (V.C.); 2 Department of Surgical, Oncological and Oral Sciences (Di.Chir.On.S.), University of Palermo, 90133 Palermo, Italy; 3 Department of Medicine and Surgery, University Hospital of Parma, 43125 Parma, Italy; capozzivitoandrea@gmail.com; 4 Department of Health Promotion, Mother and Child Care, Internal Medicine and Medical Specialties (PROMISE), University of Palermo, 90133 Palermo, Italy; 5 Department of Obstetrics and Gynecology, AOUI Verona, University of Verona, 37126 Verona, Italy; simone.garzon@univr.it

**Keywords:** gynecological oncology, locally advanced cervical cancer, conventional laparoscopy, robotic-assisted laparoscopy

## Abstract

Aortic lymph node metastases are a relative common finding in locally advanced cervical cancer. Minimally invasive surgery is the preferred approach to perform para-aortic lymph nodal staging to reduce complications, hospital stay, and the time to primary treatment. This meta-analysis (CRD42022335095) aimed to compare the surgical outcomes of the two most advanced approaches for the aortic staging procedure: conventional laparoscopy (CL) versus robotic-assisted laparoscopy (RAL). The meta-analysis was conducted according to the PRISMA guideline. The search string included the following keywords: “Laparoscopy” (MeSH Unique ID: D010535), “Robotic Surgical Procedures” (MeSH Unique ID: D065287), “Lymph Node Excision” (MeSH Unique ID: D008197) and “Aorta” (MeSH Unique ID: D001011), and “Uterine Cervical Neoplasms” (MeSH Unique ID: D002583). A total of 1324 patients were included in the analysis. Overall, 1200 patients were included in the CL group and 124 patients in the RAL group. Estimated blood loss was significantly higher in CL compared with RAL (*p* = 0.02), whereas hospital stay was longer in RAL compared with CL (*p* = 0.02). We did not find significant difference for all the other parameters, including operative time, intra- and postoperative complication rate, and number of lymph nodes excised. Based on our data analysis, both CL and RAL are valid options for para-aortic staging lymphadenectomy in locally advanced cervical cancer.

## 1. Introduction

Cervical cancer is one of the most common malignancies and is the most frequent cause of death from gynecological cancers worldwide [1]. Approximately more than one-third of patients present with locally advanced cervical cancer (LACC) at diagnosis, FIGO (International Federation of Gynecology and Obstetrics) stage IIB to IVA. This presentation is associated with 18–50% of lymph node metastases [2]. Para-aortic lymph-node status represents the most important prognostic factor in patients with LACC, with a severe negative impact on survival [3,4]. The recent FIGO 2018 classification of cervical cancer has included lymph node disease in the staging system to improve treatment allocation and inform about prognosis.

The current standard treatment in this population is concomitant chemoradiation, while the lymph node status assessment has an important staging role. The detection of para-aortic lymph node involvement is crucial to define the extent of the irradiation field and to personalize specific treatment protocols [5]. Para-aortic lymph nodal staging can be assessed using imaging, but the detection of lymph node metastases remains unsatisfactory. Even advanced imaging techniques, such as positron emission tomography-computed tomography (PET-CT), include a risk of 15% of false negative and 5–10% of false positive [6]. Computerized axial tomography (CT) and nuclear magnetic resonance (MRI) have shown lower sensitivity and specificity [7]. In this context, laparoscopic staging has been proposed as a valid alternative to the radiological nodal assessment, achieving more accurate results [8]. On the one hand, the survival benefit of surgical staging is still controversial due to conflicting results in available studies. On the other hand, the minimally invasive approach is known to improve peri- and postoperative outcomes, avoiding delay in the primary treatment. In a recent meta-analysis conducted by our group [9], we compared the two laparoscopic techniques for para-aortic surgical staging in cervical cancer: the transperitoneal laparoscopic lymphadenectomy (TLL) versus the extraperitoneal laparoscopic lymphadenectomy (ELL). Our results showed that TLL approach was associated with a higher rate of intraoperative complications, while no significant difference was found between the two techniques regarding postoperative complications. Since the introduction of the robotic surgery for gynecological oncological procedures, a significant increase of this approach has been reported following the hypothesis that the robotic route may represent an improvement compared with laparoscopic technique. Recently, the robotic approach has also been introduced for para-aortic staging in LACC patients, showing encouraging results [10].

In this scenario, the aim of our systematic review and meta-analysis was to compare the peri- and postoperative outcomes of conventional laparoscopy (CL; including either TLL and ELL) versus robotic-assisted laparoscopy (RAL) for surgical nodal assessment in LACC patients.

## 2. Materials and Methods

This systematic review was submitted in the *PROSPERO* international database (CRD42022335095) and conducted according to the PRISMA guideline (Preferred Reporting Items for Systematic Reviews and Meta-Analyses) [11]. We screened *PubMed, Scopus*, and *Web of Science* search engines from inception to March 2022. The search string included the following Medical Subject Headings (MeSH): “Laparoscopy” (MeSH Unique ID: D010535), “Robotic Surgical Procedures” (MeSH Unique ID: D065287), “Lymph Node Excision” (MeSH Unique ID: D008197) and “Aorta” (MeSH Unique ID: D001011), and “Uterine Cervical Neoplasms” (MeSH Unique ID: D002583).

Titles and/or abstracts of studies retrieved using the search strategy, and those from additional sources were screened independently by three review authors (M.C.D.D., V.G., G.L.B.) to identify studies that potentially met the aims of this systematic review. We excluded articles in languages other than English and reviews. The full text of these potentially eligible articles was retrieved and independently assessed for eligibility by other two review team members (G.S., A.A.). Any disagreement between them over the eligibility of particular articles was resolved through discussion with a third (external) collaborator. The references of the included studies were carefully evaluated to identify any potential additional source. A final review of the included articles was performed by the review supervisor (V.C.). In case of redundant articles or data used in previous studies, only the most recent articles with more comprehensive data were included in the analysis.

Prospective, retrospective, and pilot studies reporting surgical outcomes in women undergoing CL or RAL aortic lymph node dissection for surgical staging of LACC were considered and included. In case of studies reporting direct comparison between CL and RAL, each arm was separately included in the pooled analysis. Bibliographical and technical data extracted from the articles using a pre-piloted standardized form to collect the following elements: authors, publication year, the type of surgery, FIGO stage, median age and Body Mass Index (BMI), operative time (OT), estimated blood loss (EBL), hospitalization time (HT), intra- and postoperative complications, conversion to another technique, and the number of lymph nodes retrieved. The common terminology criteria for adverse events (CTCAE) grade >3 were considered for complications [12].

### Statistical Analysis

Data were expressed as standard deviation (SD) or as number (percentage). Categorical variables were compared using the chi-square or Fisher exact test. Between-group comparison of continuous variables was undertaken using the *t*-test and the Mann–Whitney nonparametric equivalent test. Two-sided *p*-values were calculated, and *p*-values < 0.05 were considered as statistically significant. Meta-analyses of proportions were used to combine data. Between-study heterogeneity was explored using the I^2^ statistic, which indicates the percentage of between-study variation that is due to heterogeneity rather than chance. A value of I^2^ of 0% indicates no observed heterogeneity, whereas values > 50% indicate a substantial level of heterogeneity. Given the small sample size of the included studies, a random effect model was preferred regardless of I^2^. StatsDirect 3.0.17 (StatsDirect Ltd., Altrincham, UK) statistical software was used for all data analyses.

## 3. Results

Eight hundred and eighty-seven studies were identified through the database search. Duplicate articles were then eliminated. After selection criteria, twenty-seven studies were considered eligible for the analysis. Twenty studies were included in the CL group (group 1) [3,10,13,14,15,16,17,18,19,20,21,22,23,24,25,26,27] and seven studies in the RAL group (group 2) [10,27,28,29,30,31]. Studies involving different types of approaches were considered as separate studies. Specifically, two studies analyzed both robotic lymphadenectomy and laparoscopic lymphadenectomy approaches [10,27], with direct comparison between the two arms. In the study by Loverix et al. [10], patients who underwent RAL had a higher American Society of Anesthesiologists score (ASA2: 62% vs. 56%, ASA3: 20% vs. 2%, *p* < 0.001), more prior major abdominal surgery (18% vs. 6%, *p* = 0.016), less EBL (median, 25 mL vs. 62.5 mL, *p* < 0.001), more para-aortic lymph nodes removed (11 vs. 6, *p* < 0.001), shorter HT (1.8 vs. 2.3 days, *p* = 0.002), and a higher but non-significant rate of metastatic para-aortic lymph nodes (13% vs. 5%, *p* = 0.065) compared with the CL, respectively; in addition, authors did not find significant differences for complication rate as well as 2-year disease-free survival (*p* = 0.472) and overall survival (*p* = 0.749) between the two approaches. Similarly, Díaz-Feijoo et al. [27] found lower EBL (90 vs. 20 mL, *p* < 0.05), and more aortic nodes were removed (14 vs. 17 nodes, *p* < 0.05) in RAL compared with CL, with an almost overlapping rate of postoperative complications (17.6% vs. 8.4%).

**Figure 1:**
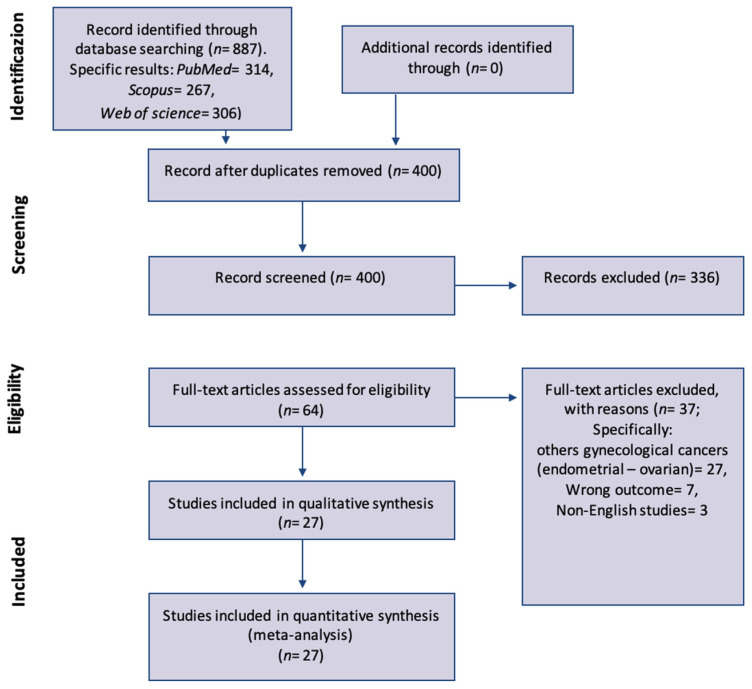
PRISMA flow-chart of study selection and inclusion.

Three studies described the two different laparoscopic approaches, TLL and ELL [14,19,26]. One study included robotic trans- and extra-peritoneal approach [29]. Furthermore, all studies were retrospective by design except one prospective randomized trial [18] and one prospective observational preliminary study [30]. The characteristics of the studies are showed in Appendix A; inclusion and exclusion criteria for each study are reported in Appendix B. The PRISMA flow chart is shown in Figure 1.

A total of 1324 patients were included in the analysis. Of these, 1200 patients were included in the CL group and 124 patients in the RAL group. The median age was 49.8 for CL and 51 for RAL. The median BMI was 25.5 and 25 for CL and RAL, respectively. The median of the OT was 129 min for patients who underwent CL aortic lymph node staging and 121.7 min for RAL aortic lymph node staging procedure. The median EBL was 81.1 mL and 26.9 mL in CL and RAL, respectively. The median length of HT was 1.9 and 3.3 days for CL and RAL, respectively. The median number of lymph nodes excised was 12.7 in the CL group and 15.7 in the RAL group. No significant differences were found between groups for BMI (*p* = 0.33), number of lymph node excised (*p* = 0.38), age (*p* = 0.62), and intraoperative time (*p* = 0.8). Conversely, EBL was significantly higher (*p* = 0.02) and HT significantly lower (*p* = 0.02) in the CL group compared with RAL group (Table 1).

**Figure 2:**
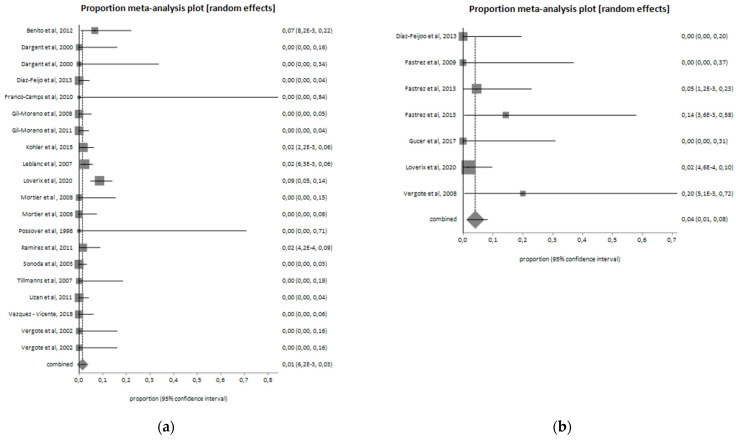
Intraoperative complications in the (**a**) laparoscopic group (I^2^ = 47.7%; pooled proportion = 1.5%) and (**b**) robotic group (I^2^ = 0%; pooled proportion = 4.1%).

Intraoperative complications were reported in 23 patients (2%) in the CL group and in 4 patients (3.2%) in the RAL group. The most frequent intraoperative complications were vascular and urinary ones. The type of intraoperative complications occurred in the two groups are shown in Table 2. As shown in Figure 2, the intraoperative complications pooled proportion is 4.1% (I^2^ = 0%) for the RAL and 1.5% (I^2^ = 47.7%) for CL.

**Figure 3:**
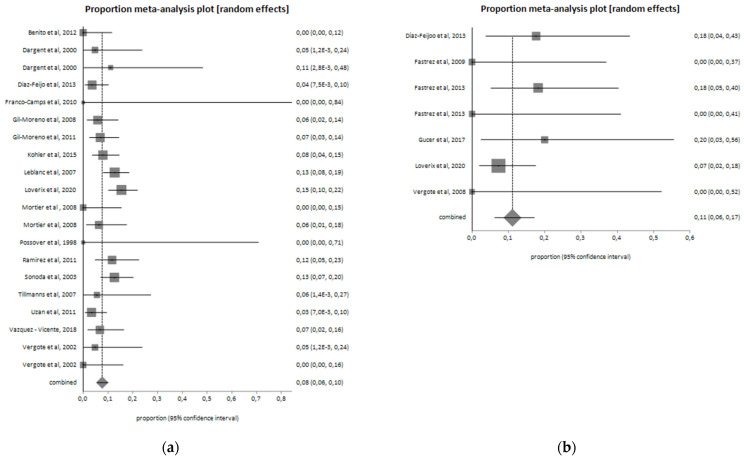
Postoperative complications in the (**a**) laparoscopic group (I^2^ = 43.9%; pooled proportion = 7.7%) and (**b**) robotic group (I^2^ = 0%; pooled proportion = 11.1%).

**Figure 4:**
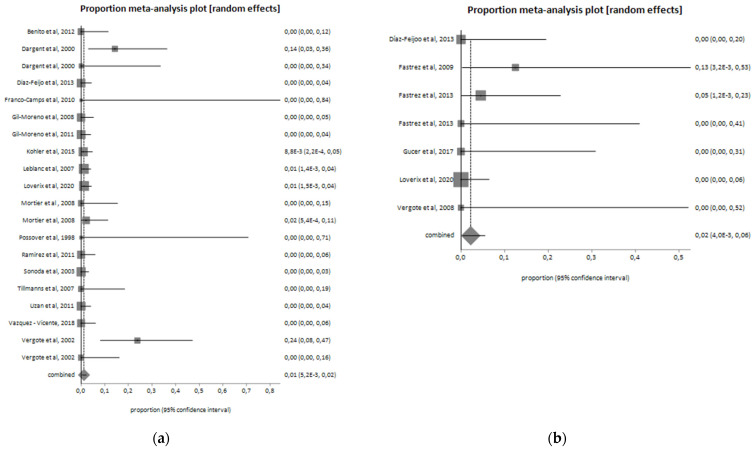
Conversion from minimally invasive to open surgery (laparotomy) in the (**a**) laparoscopic group (I^2^ = 38.4%; pooled proportion = 1.2%) and (**b**) robotic group (I^2^ = 0%; pooled proportion = 2.2%).

In total, 104 patients (8.6%) of the CL group developed postoperative complications, while 13 patients (9.7%) of the RAL group reported a complication after surgery. Postoperative complications are reported in Table 3. In the two groups, sixty-three lymphatic complications occurred, ten to urinary compartment, three trocar site hernia, and one bowel complication. The postoperative complications pooled proportion is 11.1% (I^2^ = 0%) for the RAL group and 7.7% (I^2^ = 43.9%) for CL. Pooled proportion of post-operative complications among the two groups are shown in Figure 3. In 14 (1.2%) cases of the CL group and in 2 (1.6%) of the RAL group, a conversion to laparotomy was required (Figure 4).

## 4. Discussion

Aortic lymph-node metastases are common findings in LACC patients, reaching a rate of about 40–70% for stages III and IV, and the diagnostic value of surgical para-aortic lymph node dissection has been widely investigated [32]. Although the increased morbidity is associated with surgical procedures, minimally invasive surgery (MIS) for surgical staging in cervical cancer provides several advantages without compromising the course of the disease [3]. Reduction in peri- and postoperative complications and reduction of HT represent the main benefits of this surgical approach. Moreover, MIS allows to perform para-aortic lymphadenectomy even in the difficult case of abdominal vascular and urinary anomalies [33].

Considering the only staging intent, in order to avoid delay of the primary chemoradiation, it is essential to propose the most effective and technologically advanced surgical approach. Laparoscopic aortic staging is one of the most challenging procedures in gynecologic oncology: in this scenario, RAL can have advantages over CL with faster learning curve, technical improvements such as a 3D imaging, elimination of physiological tremor, and increased precision due to the seven-degree instrument’s articulation [34]. Accumulating evidence suggests the overall feasibility of robotic para-aortic lymph node staging for cervical cancer [10,27]; in addition, some authors have compared the RAL surgical staging to the CL and have found better perioperative outcomes and similar survival outcomes [10,27]. Our meta-analysis showed that RAL surgical staging in LACC patients is significantly associated with less EBL than conventional laparoscopy. Furthermore, OT and the number of lymph nodes excised are in favor of robotic approach although the difference is not statistically significant. Similar to previous series, our data analysis supports RAL as an appropriate alternative to CL for para-aortic lymph node dissection in LACC patients.

Although not significant, the higher number of lymph nodes excised using RAL compared with CL could be due, at least in part, to the greater precision of the robotic procedure and the possibility of being more radical with fine dissection in difficult anatomical spaces. Interestingly, we found shorter HT for the CL group compared with RAL: although we cannot explain with absolute certainty this data, this is probably due to the type of women who are addressed to RAL; indeed, patients undergoing robotic approach usually have more comorbidities (e.g., obese, more prior abdominal surgery, higher ASA score) [10], and this may be associated with longer HT.

Lymphatic complications represent the most frequent postoperative complication, especially for the robotic group. One possible and reliable explanation of this difference is related to using different instruments in the two surgical routes. During a laparoscopic lymphadenectomy, multifunction instruments seal and cut the lymphatic vessels, unlike the robotic approach, in which bipolar energy and scissors are commonly used. Moreover, some authors suggest systematically clipping any large lymphatic vessel to avoid lymphatic complications [3,16]. Vascular injuries and ureteral lesions were the most frequent intraoperative complications, especially in the robotic group. On the one hand, some authors suggested that during RAL, pneumoperitoneal pressure is lower than laparoscopy, and very often, the patients selected for RAL are already subjected to previous surgery, obese, and with comorbidities [35,36]: this challenging surgical scenario may limit the visualization of the ureter and the great vessels, increasing intraoperative complications [34,35]. On the other hand, the RAL approach allows a better exposition of the pre-cava and inter-aortocaval field than the CL. Indeed, robotic surgery is associated with a higher Trendelenburg inclination and a better range of motion of the instruments [10,27]. In addition, the learning curve for laparoscopic aortic lymphadenectomy [37] is longer than robotic procedure [38] due to limited rigid instruments and the 2-dimensional view of the laparoscope’s video camera, which requires greater surgeon skills to perform this procedure. Furthermore, aortic lymphadenectomy is a single-quadrant surgery and perfectly matches with the robotic approach, using its increasing precision that would otherwise suffer in case of re-docking [39]. However, the selection of the patients for lymph node staging surgery should be careful, considering that the benefits of diagnostic surgery should justify its possible morbidities.

Limits of the present meta-analysis are represented by the small sample size of robotic group, the heterogenicity of the studies included, the retrospective nature of most of the articles analyzed. In addition, most of the included studies reported insufficient information on the pre-operative characteristics of the patients, which did not allow us to perform a robust sub-analysis (meta-regression) of surgical outcomes based on these parameters. Finally, only one study reported either the transperitoneal or the extra peritoneal technique for the robotic aortic lymph node dissection. However, good heterogenicity of the studies is showed by the pooled analysis.

## 5. Conclusions

Based on our data analysis, both CL and RAL can be considered valid options for para-aortic staging lymphadenectomy in women with LACC, with comparable safety and surgical outcomes. In particular, the two techniques allowed similar operative time, intra- and postoperative complication rate, and number of lymph nodes excised.

## Figures and Tables

**Table 1:** Analysis of surgical outcomes between conventional laparoscopy and robotic groups.

	Laparoscopic	Robotic	*p*
Number of studies	20	7	
Number of cases	1200	124	
Operative time (min)	129	121.7	0.8
Total complications (*n*)	127	17	0.29
Intraoperative complications (*n*)	23	4	0.31
Postoperative complications (*n*)	104	13	0.5
Number of lymph nodes excised	12.7	15.7	0.38
EBL	81.1	26.9	0.02
Hospital stay	1.9	3.3	0.02
Age	49.8	51.0	0.62
BMI	25.5	25.0	0.33

Min, minutes; EBL, estimated blood loss; BMI, body mass index.

**Table 2:** Analysis of intraoperative complications in conventional laparoscopy and robotic groups.

Type of Intraoperative Complication	Laparoscopic (*n* = 1200)	Robotic (*n* = 124)	*p*
Vascular injuries	18 (1.5%)	2 (1.6%)	
Ureteric injuries	3 (0.3%)	2 (1.6%)	
Nerve injury	1 (0.1%)	0 (0%)	
Bowel injury total	1 (0.1%)	0 (0%)	
Total	23 (2%)	4 (3.2%)	0.31

**Table 3:** Analysis of postoperative complications in conventional laparoscopy and robotic groups.

Type of Postoperative Complication	Laparoscopic (*n* = 1200)	Robotic (*n* = 124)	*p*
Lymphatic complication	57 (4.7%)	6 (4.8%)	
Vascular complication	15 (1.2%)	0 (0%)	
Urinary complication	7 (0.6%)	3 (2.4%)	
Bowel complication	1 (0.1%)	0 (0%)	
Trocar site hernia	2 (0.2%)	1 (0.8%)	
Others	22 (1.8%)	3 (2.4.%)	
Total	104 (8.6%)	13 (10.4%)	0.5

## Data Availability

Data analyzed in this systematic review and meta-analysis were already available in the single studies included.

## Appendix A

**Table A1:** Characteristics of the included studies.

Authors, Years	Type of Study	Cases	Stage	OT (Minutes)	EBL (mL)	Conversions	HT (Days)	Number of Lymph Nodes Excised	Intra-Operative Complications (n and Type)	Post-Operative Complications (n and Type)	BMI (Median)	Age (Median)	Technique
Díaz-Feijoo et al., 2013 [27]	Retrospective study	17	IB2-IVA	150	20	0	2	17	0	3 (not specified)	23	49	Robotic Retroperitoneal
Fastrez et al., 2009 [28]	Retrospective study	8	IB2-IVA	137.5	/	1	4.5	14	0	0	24.3	58	Robotic Transperitoneal
Fastrez et al., 2013 [29]	Retrospective study	22	IB2-IVA	165	/	1	6	19.5	1 aortic injury	4: 1 chylous ascites; 2 symptomatic lymphocele; 1 epiploic hernia through umbilical port	27	55	Robotic Transperitoneal
Fastrez et al., 2013 [29]	Retrospective study	7	IB2-IVA	100	/	0	2.5	9.5	1 partial section of the right ureter	0	24	50.5	Robotic Retroperitoneal
Gucer et al., 2017 [30]	Prospective observational preliminary study	10	IIB-IVA	141	12.5	0	4	25	0	2: 1 symptomatic lymphocyst; 1 local infection on assistant port site	28.5	46	Robotic Transperitoneal
Loverix et al., 2020 [10]	Retrospective study	55	IB1-IVA	74.5	25	0	1.8		1 bleeding	4: 3 urinary tract infection; 1 salpingitis	24.7	49	Robotic Transperitoneal and Retroperitoneal
Vergote et al., 2008 [31]	Retrospective study	5	IIB-IIIB	83.8	50	0	2.2	9.2	1 right ureter damage	0	23.8	49.6	Robotic Retroperitoneal
Benito et al., 2012 [13]	Retrospective study	30	IB2-IVA	118.7	75	0	1.9	14.2	2: 1 lumbar artery injury; 1 bowel injury	0	26.3	47.6	Laparoscopic Retroperitoneal
Dargent et al., 2000 [14]	Retrospective study	21	IB1-IVA	119	/	3	/	15	0	1 lymphocele	23	50	Laparoscopic Retroperitoneal
Dargent et al., 2000 [14]	Retrospective study	9	IB1-IVA	160	/	0	/	19	0	1 phlebitis	23	50	Laparoscopic Transperitoneal
Diaz-Feijoo et al., 2013 [27]	Retrospective study	83	IB2-IVA	150	20	0	2	17	0	3: 2 lymphocysts; 1 chylous ascites	26.4	51	Laparoscopic Retroperitoneal
Franco-Camps et al., 2010 [15]	Retrospective study	2	IIIB-IVA	140	95	0	2	6	0	0	29	71	Laparoscopic Retroperitoneal
Gil-Moreno et al., 2008 [17]	Retrospective study	69		140	100	0	2	15.2	0	4: 2 retroperitoneal hematoma; 2 lymphocyst	27	51	Laparoscopic Retroperitoneal
Gil-Moreno et al., 2011 [16]	Retrospective study	87	IB2-IVA	150		0	2		0	6: 2 retroperitoneal hematoma; 3 lymphocysts;1 urinary tract infection	26.5		Laparoscopic Retroperitoneal
Köhler et al., 2015 [18]	Trial	113	IIB-IVA	/	/	1	/	17	2 vascular injuries	9: 1 thrombosis; 1 ileus; 4 symptomatic lymphoceles; 1 nerve irritation; and 2 others (not specified)	26.2	47.2	Laparoscopic Transperitoneal
Leblanc et al., 2007 [3]	Retrospective study	173	IB2-IVA	155	100	2	1.4	20.8	4: 1 obturator nerve injury; 2 ureteric injuries; 1 vascular injury (vena cava)	22: 17 symptomatic lymphocysts; 3 transient ascites; 1 retroperitoneal hematoma; 1 bowel obstruction resulting from herniating bowel through an umbilical port site	27.1	45	Laparoscopic Retroperitoneal
Loverix et al., 2020 [10]	Retrospective study	162	IIB-IVA	75	62.5	2	2		14: 2 ureteral trauma;11 bleeding; 1 other	25: 2 wound problem with conservative management; 1 retroperitoneal hematoma with conservative management; 1 severe pain; 4 urinary tract infection;8 blood transfusion; 1 vasovagal syncope; 1 severe vaginal blood; 1 iron supplements for anemia; 1 DVT with lung embolism treated with LMWH; 1 placement of ureteral stent for ureteral trauma; 1 retroperitoneal abscess with evacuation under anesthesia; 1 re-laparotomy for bleeding of the internal epigastric artery; 1 laparoscopic repair of ureteral trauma; 1 hospitalization in intensive care unit for hyponatremia	24.4	48	Laparoscopic Transperitoneal and Retroperitoneal
Mortier et al., 2008 [19]	Retrospective study	22	IB2-IIIB	68	90	0	2	5	0	0	24	48	Laparoscopic Transperitoneal
Mortier et al., 2008 [19]	Retrospective study	47	IB2-IIIB	62	90	1	2	8	0	3: 2 lymphocoeles; 1 retroperitoneal hematoma	24	48	Laparoscopic Retroperitoneal
Possover et al., 1998 [20]	Retrospective study	3	IIIB	218	200	0	4	10	0	0	/	46.3	Laparoscopic Retroperitoneal
Ramirez et al., 2011 [21]	Retrospective study	60	IB2-IVA	140	22.5	0	1	11	1 bleeding from an ascending lumbar vein at the level of the left renal vein	7 lymphocyst	26,7	48	Laparoscopic Retroperitoneal
Sonoda et al., 2003 [22]	Retrospective study	111	IB2-IVA	157	100	0	2	19	0	14: 11 symptomatic lymphoceles; 2 retroperitoneal hematomas; 1 trocar-site hernia	24	46	Laparoscopic Retroperitoneal
Tillmanns et al., 2007 [23]	Retrospective study	18	IIB-IVA	108	25	0		10	0	1 lymphocyst	29	49	Laparoscopic Retroperitoneal
Uzan et al., 2011 [24]	Retrospective study	89	IB2-IVA	185	/	0	3	13	0	3 lymphocysts	23	45	Laparoscopic Retroperitoneal
Vázquez-Vicente, 2018 [25]	Retrospective study	59	IB2-IVA	180	/	0	1.7	16.4	0	4: 3 lymphoceles; 1 intrabdominal abscess	24.6	52.3	Laparoscopic Transperitoneal and Retroperitoneal
Vergote et al., 2002 [26]	Retrospective study	21	IB2-IIIB	55	78	5	1	6	0	1 retroperitoneal hematoma	/	51	Laparoscopic Retroperitoneal
Vergote et al., 2002 [26]	Retrospective study	21	IB2-IIIB	70	78	0	1	6	0	0	/	51	Laparoscopic Transperitoneal

OT, operative time; EBL, estimated blood loss; BMI, body mass index; HT, Hospitalization Time.

## Appendix B

**Table A2:** Inclusion and exclusion criteria of the studies.

Author, Year	Inclusion Criteria	Exclusion Criteria
Díaz-Feijoo et al., 2013 [27]	- Patients with locally advanced cervical cancer (FIGO stages IB2–IVA) who underwent robotic-assisted laparoscopic extraperitoneal paraaortic lymphadenectomy	- Severe cardiorespiratory disease - Age ≥ 80 years - Prior radiotherapy - Prior retroperitoneal surgery - Evidence of metastatic disease outside of the pelvis in preoperative imaging study
Fastrez et al., 2009 [28]	- Patients with locally advanced cervical cancer (FIGO stages IB2–IVA) who underwent robotic-assisted laparoscopic transperitoneal paraaortic lymphadenectomy	/
Fastrez et al., 2013 [29]	- Patients with locally advanced cervical cancer (FIGO stages IB2–IVA) - Patients with early-stage disease (FIGO IA1–IB1) who had histologically proven positive pelvic LNs - Patients underwent robotic-assisted laparoscopic extraperitoneal paraaortic lymphadenectomy	/
Gucer et al., 2017 [30]	- Patients with locally advanced cervical cancer (FIGO stages IIB–IVA) who underwent robotic-assisted laparoscopic transperitoneal paraaortic lymphadenectomy	- Severe cardiorespiratory disease - Age ≥ 70 years - Prior to radiotherapy - Prior surgery in the retroperitoneal para-aortic area - Evidence of metastatic disease outside of the pelvis in imaging studies
Loverix et al., 2020 [10]	- Patients with locally advanced cervical cancer (FIGO stage IB2–IVA or IB1 with suspicious pelvic lymph nodes) who underwent a para-aortic lymphadenectomy up to the inferior mesenteric artery	- Simultaneous presence of other primary malignancies - Para-aortic lymphadenectomy combined with other surgery (such as hysterectomy, pelvic lymphadenectomy or omentectomy - Prior radiotherapy or retroperitoneal surgery - Metastatic disease outside of the pelvis on preoperative imaging - Poor general condition of the patient - Inoperability due to intraperitoneal adhesions
Vergote et al., 2008 [31]	- Patients with locally advanced cervical cancer (FIGO stages IIB–IIB) who underwent robotic-assisted laparoscopic extraperitoneal paraaortic lymphadenectomy - No evidence of disease outside the pelvis	/
Benito et al., 2012 [13]	- Patients with locally advanced cervical cancer (stages IB2–IVA) or enlarged pelvic lymph nodes on a preoperative CT scan (>1 cm) who underwent laparoscopic extraperitoneal para-aortic lymphadenectomy - Absence of pathological nodes at the para-aortic level - Laparoscopic surgery not contraindicated	/
Dargent et al., 2000 [14]	- Patients with cervical cancer who underwent extraperitoneal laparoscopic paraaortic lymphadenectomy	/
Franco-Camps et al., 2010 [15]	- Patients with cervical cancer - Patients with probable isolated nodal recurrence in the paraaortic territory by CT, MRI, or PET - Lymph nodes were considered pathologic if they measured >1 cm at their maximum short axis in CT scanning or MRI	/
Gil-Moreno et al., 2008 [17]	- Patients with bulky and locally advanced cervical carcinoma (FIGO stages IB2, IIA > 4 cm, and IIB–IVA), without evidence of distant spread, who underwent extraperitoneal laparoscopic lymphadenectomy of the common pelvic and para-aortic lymph nodes for surgical staging	/
Gil-Moreno et al., 2011 [16]	- Patients with bulky or locally advanced cervical cancer (FIGO stages IB2, IIA2, and IIB-IVA), without evidence of distant spread, who underwent extraperitoneal laparoscopic infrarenal aortic and common iliac dissection for surgical staging	- Severe cardiorespiratory disease - Age ≥ 80 years - Prior radiotherapy or retroperitoneal surgery - Evidence of metastatic disease outside of the pelvis
Köhler et al., 2015 [18]	- Histological reports confirming the presence of squamous cell carcinoma, adenocarcinoma, or adenosquamous cervical cancer - FIGO stage ranging from IIB to IVA. - Pretreatment imaging included a whole abdominal CT and/or an abdominal MRI and/or PET-CT as well as chest imaging - Patients underwent surgical staging with a transperitoneal laparoscopic or extraperitoneal laparoscopic approach	/
Leblanc et al., 2007 [3]	- Patients with locally advanced cervical cancer (FIGO stages IB2–IVA) - No evidence of extrapelvic disease at preoperative imaging (MRI and/or CT scan) - Age < 70 years - Patients with a recurrent pelvic cervical carcinoma, candidates for an exenterative procedure were submitted to the same procedure	/
Mortier et al., 2008 [19]	- Patients with cervical carcinoma with clinical FIGO stage IB2–IIIB who underwent a laparoscopic retroperitoneal para-aortic lymphadenectomy as staging procedure	- Coagulation disorders - Presence of metastatic para-aortic lymph nodes on PET/PET-CT and/or CT (preoperative presence of metastatic nodes was defined as para-aortic lymph nodes larger than 1 cm with uptake of contrast on CT and/or PET scan)
Possover et al., 1998 [20]	- Patients with advanced cervical cancer who underwent extraperitoneal laparoscopic suprarenal para-aortic lymph node sampling	/
Ramirez et al., 2011 [21]	- Patients with advanced cervical cancer stage IB2–IVA cervical cancer - Biopsy-proven cervical carcinoma, any histology - No evidence of para-aortic lymphadenopathy (all nodes <2 cm in diameter) on a preoperative CT or MRI scan of the abdomen and pelvis - Adequate bone marrow, renal, and hepatic function - Zubrod Performance Status of 0, 1, or 2	- Prior retroperitoneal surgery - Prior pelvic or abdominal radiotherapy - Upper abdominal intraperitoneal disease - Evidence of ovarian metastases - Pregnant patients - Evidence of distant metastases on imaging studies or physical examination - Patients with contraindications to laparoscopy
Sonoda et al., 2003 [22]	- Patients with locally advanced cervical cancer (FIGO stages IB2–IVA) who underwent a laparoscopic extraperitoneal paraaortic and common iliac lymph node dissection	- Radiographic enlarged or cytologically positive paraaortic nodes
Tillmanns et al., 2007 [23]	- Patients with locally advanced cervical cancer (FIGO stages IB2–IVA) who underwent extraperitoneal para-aortic lymphadenectomy	- Pelvic nodal disease > 1.5 cm on pre-operative CT scan - Enlarged aortic nodal disease (>1.0 cm) on pre-operative CT scan
Uzan et al., 2011 [24]	- Patients with locally advanced cervical cancer (FIGO stages IB2–IVA) who underwent a extraperitoneal para-aortic lymphadenectomy - Age < 70 years	- Evidence of extrapelvic disease on preoperative imaging (MRI or CT scan)
Vázquez-Vicente, 2018 [25]	- Patients with locally advanced cervical cancer (FIGO stages IB2–IVA), who underwent extraperitoneal para-aortic lymphadenectomy	/
Vergote et al., 2002 [26]	- Patients with primary cervical carcinoma stage IB2–IIIB who underwent laparoscopic lower para-aortic lymphadenectomy	- Patients with suspicious para-aortic nodes on CT scan

FIGO, International Federation of Obstetrics and Gynecology; LN, lymph node; CT, computed tomography; MRI, magnetic resonance imaging; PET, positron emission tomography.
